# Rab6a is a novel regulator of meiotic apparatus and maturational progression in mouse oocytes

**DOI:** 10.1038/srep22209

**Published:** 2016-02-26

**Authors:** Xiaojing Hou, Jiaqi Zhang, Ling Li, Rujun Ma, Juan Ge, Longsen Han, Qiang Wang

**Affiliations:** 1 State Key Laboratory of Reproductive Medicine, Nanjing Medical University, Nanjing, China; 2 Center of Reproductive Medicine, Jinling Hospital, Medical School of Nanjing University, Nanjing, China

## Abstract

Rab family GTPases have been well known to regulate intracellular vesicle transport, however their function in mammalian oocytes has not been addressed. In this study, we report that when Rab6a is specifically knockdown, mouse oocytes are unable to progress normally through meiosis, arresting at metaphase I. Moreover, in these oocytes, the defects of chromosome alignment and spindle organization are readily observed during maturation, and resultantly increasing the aneuploidy incidence. We further reveal that kinetochore-microtubule attachments are severely compromised in Rab6a-depleted oocytes, which may in part mediate the meiotic phenotypes described above. In addition, when Rab6a function is altered, BubR1 levels on the kinetochores are markedly increased in metaphase oocytes, indicating the activation of spindle assembly checkpoint. In sum, we identify Rab6a as an important player in modulating oocyte meiosis, specifically the chromosome/spindle organization and metaphase-anaphase transition.

To ensuring successful fertilization and early embryonic development, the oocyte undergoes specialized cell divisions named meiosis I and II. Meiosis I begins with germinal vesicle breakdown (GVBD) after stimulation by pituitary luteinizing hormone (LH) and ends with first polar body (PB1) extrusion^1^,^2^. During meiosis, microtubules first organize into a barrel-shaped bipolar spindle, with all chromosomes aligned at the metaphase plate, following which recombined homologous chromosomes are segregated at anaphase/telophase^3^. Oocytes are finally progress uninterruptedly to meiosis II and become arrested for a second time waiting for fertilization^4^. Notably, it has been suggested that chromosome segregation is error prone during mammalian oocyte meiosis^5^,^6^. Any mistakes in this process can result in the generation of aneuploid embryos, contributing to pregnancy loss or severe birth defects^7^. Similar to mitosis, spindle assembly checkpoint (SAC) mechanism in oocytes prevents the premature metaphase-anaphase transition until all chromosomes successfully attach to the bipolar spindle with proper tension^8^,^9^. The core components of SAC are the Mad and Bub protein families, which inhibit the activation of the anaphase promoting complex (APC) and therefore the degradation of cyclin B and anaphase onset^10^.

Rab (Ras-related proteins in brain) GTPases are evolutionarily conserved, essential components of vesicle trafficking pathways. Over 70 human Rab and Rab-like members of the Ras superfamily have been identified^11^. For example, Rab1, which is located at endoplasmic reticulum (ER) exit sites and the pre-Golgi intermediate compartment, mediates ER–Golgi trafficking^12^. Rab5, localized to early endosomes and phagosomes, has been well recognized to involve in membrane tethering and docking^13^,^14^. Most recent findings have also suggested that Rab5 GTPase participates in chromosome congression and spindle assembly in both mitotic cells and meiotic oocytes^15^,^16^. Two isoforms of Rab6 GTPase, Rab6a′ and Rab6a, differ in only three amino acids and are expressed in mammalian cells^17^,^18^. Rab6 has been shown to regulate a retrograde transport route connecting early endosomes to ER^18^,^19^. Rab6a′ functions in a pathway involved in mitotic arrest through the interaction with dynein/dynactin complex at the kinetochores^20^. During *Drosophila* oogenesis, Rab6 is required for the polarization of the oocyte microtubule cytoskeleton and for the posterior localization of oskar mRNA^21^. However, so far, the function of Rab6 during mammalian oocyte meiosis remains unknown.

Here, by employing siRNA knockdown analysis, we discovered the involvement of Rab6a in meiosis of mouse oocyte, particularly in controlling meiotic progression and meiotic structures, and report our findings below.

## Results

### Rab6a knockdown adversely affects maturational progression of mouse oocytes

**Figure 1:**
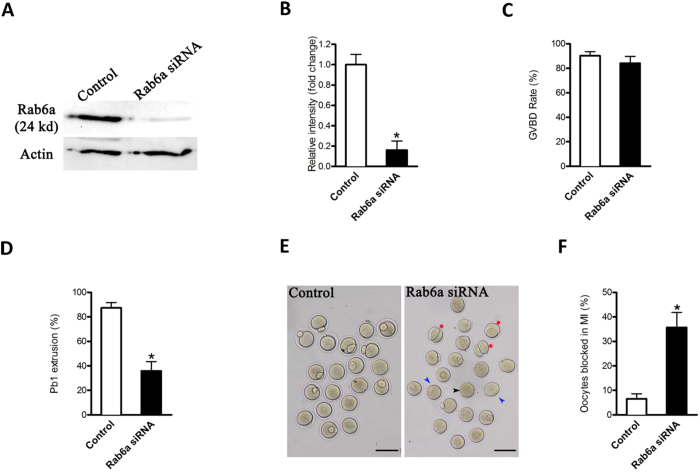
Effects of Rab6a knockdown on oocyte maturation. Fully-grown oocytes were injected with Rab6a-siRNA, arrested at GV stage with milrinone for 20 hours to allow mRNA degradation, and then cultured in milrinone-free medium to evaluate the maturational progression. (**A**) Western blot showing the reduced expression of Rab6a after siRNA injection. (**B**) Band intensity was measured using Image J software, and the ratio of Rab6a/Actin expression was normalized. (**C,D**) Quantitative analysis of GVBD rate and Pb1 extrusion rate in control (n = 150) and Rab6a-siRNA (n = 132) oocytes. (**E**) Phase-contrast images of control siRNA injected and Rab6a knockdown oocytes. Blue arrowheads point to oocytes that fail to extrude a polar body; red asters denote oocytes with apparent symmetrical division. (**F**) Percentage of oocytes blocked in metaphase after Rab6a-siRNA injection. The graph shows the mean ± SD of the results obtained in three independent experiments, in which at least 90 oocytes were analyzed. Scale bars, 100 μm. *p < 0.05 vs control.

To explore the function of Rab6a, fully-grown oocytes were injected with specifically-designed siRNA (Rab6a-siRNA); a negative control siRNA was included as control. After microinjection, the oocytes were arrested at GV stage for 20 hours with milrinone to promote mRNA degradation. Immunoblotting confirmed that the significant reduction of Rab6a proteins in oocytes (Fig. 1A,B). After 3 hours culture, both control and Rab6a-knockdown groups resumed meiosis normally, indicated by the similar GVBD rate (Fig. 1C). However, Pb1 extrusion was decreased in Rab6a-siRNA oocytes compared to controls (35.9 ± 7.6% vs. 87.4 ± 4.3% control, *p* < 0.05; Fig. 1D), indicating that Rab6a-depleted oocytes failed to complete meiosis I and form the first polar body (Fig. 1E, blue arrowheads). In some oocytes where a meiotic division appears to have been completed, the symmetrical division was frequently observed (Fig. 1E, red asters). To further define the developmental stage of those Rab6a-siRNA oocytes without polar body, we performed nuclear staining and quantitative analysis. As shown in Fig. 1F, we found that more than 35% of oocytes injected with Rab6a-siRNA were blocked in meiosis I, which was dramatically increased compared to controls. Together, these results suggest that alteration of Rab6a function adversely impacts oocyte maturation and meiotic divisions.

### Rab6a functions in chromosome alignment and spindle organization

**Figure 2:**
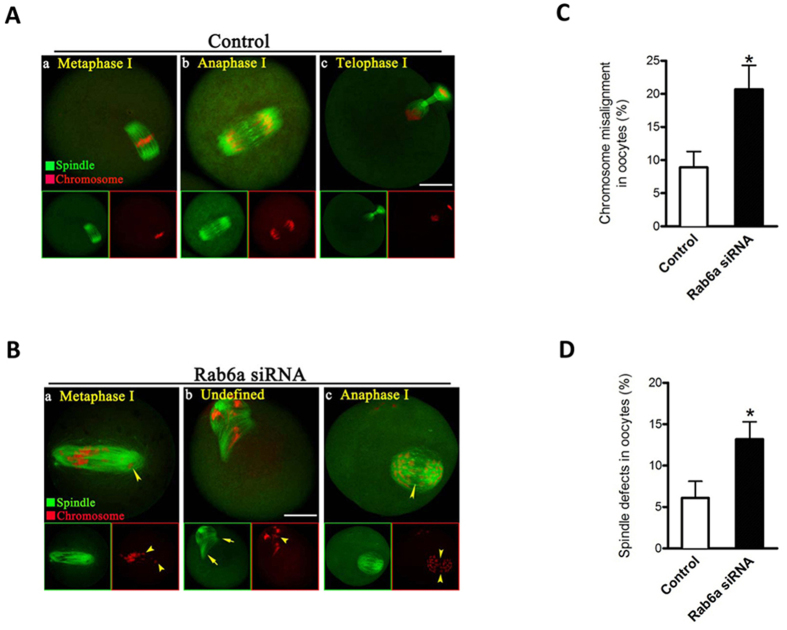
Rab6a depletion causes spindle disorganization and chromosome misalignment in oocyte meiosis. Control and Rab6a-siRNA oocytes were stained with α-tubulin antibody to visualize the spindle (green) and counterstained with PI to visualize chromosomes (red). (**A**) Control metaphase oocytes (a) present a typical barrel-shape spindle and well-aligned chromosomes on the metaphase plate; (b) in anaphase oocytes (b) chromosomes move evenly away from the equator toward opposite poles, and homologous chromosomes are accurately segregated in telophase oocytes (c). (**B**) Spindle defects (arrows) and chromosomes misalignment (arrowheads) were frequently observed in Rab6a-depleted oocytes. Representative confocal sections are shown. Scale bar, 25 μm. (**C**) Quantification of control and Rab6a-siRNA oocytes with chromosome misalignment. (**D**) Quantification of control and Rab6a-siRNA oocytes with spindle defects. Data are expressed as mean percentage ± SD from three independent experiments in which at least 100 oocytes were analyzed. *p < 0.05 vs. controls.

The above data prompted us to ask whether depletion of Rab6a affects the meiotic apparatus in oocytes. For this purpose, Rab6a-siRNA and control oocytes were immunolabeled with anti-tubulin antibody to visualize the spindle and co-stained with propidium iodide (PI) for chromosomes. Confocal microscopy revealed that most control oocytes at metaphase presented with a typical barrel-shape spindle and well-aligned chromosomes on the metaphase plate (Fig. 2Aa). In striking contrast, we found a high percentage of chromosome congression failure (20.7 ± 3.6% vs. 8.9 ± 2.4% control, *p* < 0.05; Fig. 2C) and spindle defects (13.2 ± 2.1% vs. 6.1 ± 2.0% control, *p* < 0.05; Fig. 2D) in Rab6a-depleted oocytes, showing diverse disorganized spindles (Fig. 2Ba,b, arrows) with several scattered chromosomes (Fig. 2Ba,b, arrowheads).

As shown in Fig. 2Ab,c, during normal oocyte meiosis I, accompanying with chromosomes moving evenly away from the equator toward opposite poles, homologous chromosomes are accurately segregated at anaphase and telophase stages. By contrast, in a small number of anaphase/telophase oocytes depleted of Rab6a, the aberrant chromosome separation was readily detected (Fig. 2Bc, arrowheads), a lagging chromosome phenotype that was about 4 times more prevalent than in control oocytes. Taken together, these results indicate that Rab6a is required for chromosome movement and spindle assembly in meiotic oocytes.

### Increased incidence of aneuploidy in Rab6a-depleted oocytes

**Figure 3:**
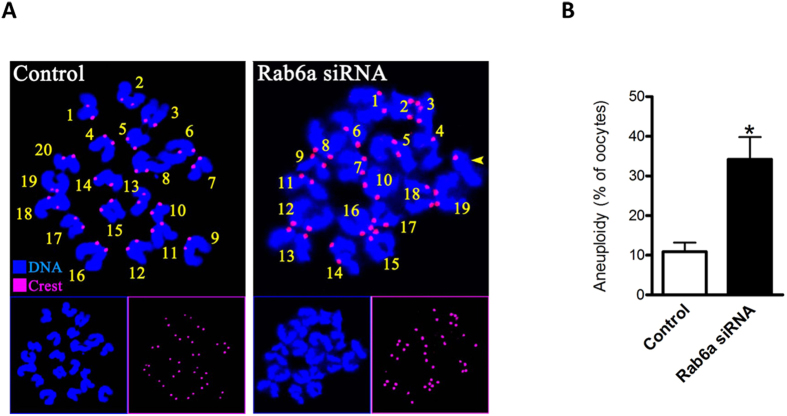
Increased incidence of aneuploidy in Rab6a-siRNA oocytes. (**A**) Chromosome spread of control and Rab6a-siRNA MII oocytes. Chromosomes were stained with Hoechst 33342 (blue) and kinetochores were labeled with CREST (purple). Representative confocal images indicate euploid control oocytes, and aneuploid Rab6a-siRNA oocytes with 19 chromosomes and one chromatid (yellow arrowhead). (**B**) Quantification of aneuploidy in control and Rab6a-siRNA oocytes. 35 control oocytes and 30 Rab6a-siRNA oocytes were analyzed respectively. Error bars indicate ± SD. *p < 0.05 vs. controls.

Since Rab6a knockdown led to the chromosome misalignment and missegregation during meiosis, we postulated that the numerical abnormalities of chromosomes may be induced in matured Rab6a-siRNA oocytes. To test this hypothesis, we analyzed the karyotype of MII oocytes by chromosome spreading combined with kinetochore labeling. As shown in Fig. 3, aneuploidy was observed in 34.2% of oocytes injected with Rab6a-siRNA compared with 10.5% of controls (Fig. 3A shows representative images of euploidy and aneuploidy, respectively; Fig. 3B). These observations suggest that loss of Rab6a disrupts the assembly of meiotic spindle and movement of meiotic chromosomes, consequently contributing to the generation of aneuploid eggs.

### Rab6a knockdown impairs the kinetochore-microtubule attachments in oocyte

Next, we decided to search for a potential mechanism that would explain the requirement of Rab6a for meiotic regulation in oocytes. On meiotic entry, dynamic microtubules form a bipolar spindle, which is responsible for capturing and congressing chromosomes. These events require proper attachment between spindle microtubules and kinetochores, large protein structures built on centromeric chromatin^22^. We therefore asked whether kinetochore-microtubule (K-MT) attachments are defective in Rab6a-depleted oocytes. To do this, metaphase I oocytes were immunolabeled with CREST to detect kinetochores, with anti-tubulin antibody to track microtubules as described previously^15^.

**Figure 4:**
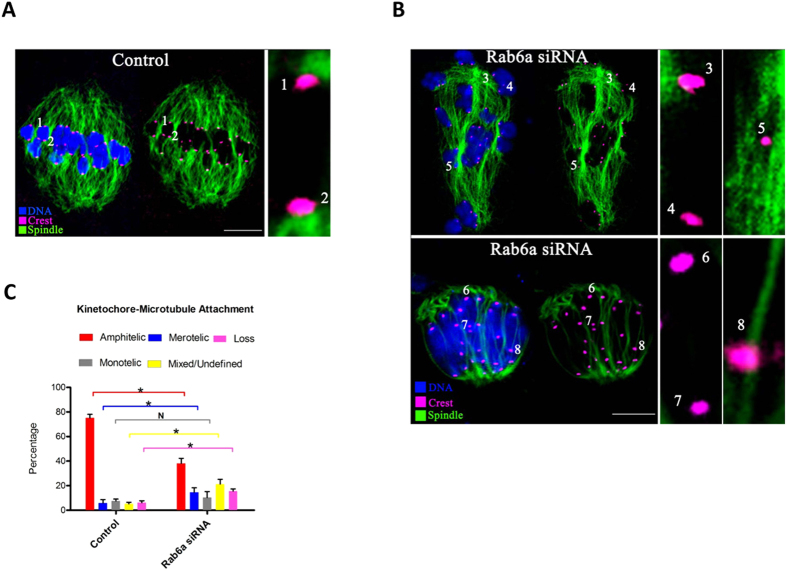
Rab6a-depleted oocytes display impaired kinetochore-microtubule attachments. (**A**) Control and Rab6a-siRNA oocytes at MI stage were labeled with α-tubulin antibody to visualize spindle (green), CREST to detect kinetochore (purple), and co-stained with Hoechst 33342 for chromosomes (blue). (**A**) Representative confocal sections showing the amphitelic attachment in control oocyte (Chromosome 1 and 2). (**B**) Representative confocal sections showing the monotelic attachment (Chromosome 3 and 4), mixed/undefined attachment (Chromosome 5), loss attachment (Chromosome 6 and 7), and merotelic attachment (Chromosome 8) in Rab6a-siRNA oocytes. (**C**) Quantitative analysis of K-MT attachments in oocytes as indicated. Kinetochores in regions where fibers were not easily visualized were not included in the analysis. 15 control oocytes and 12 Rab6a-siRNA oocytes were examined respectively. Scale bars, 15 μm. *p < 0.05 vs. controls.

By confocal microscopy we found that the predominant pattern of K-MT in normal oocytes is amphitelic attachment, in which the kinetochore of one chromosome is connected to the spindle pole and the kinetochore of the other chromosome is connected to the opposite spindle pole (Fig. 4A, chromosomes 1 and 2). Of note, quantitative analysis revealed that the proportion of amphitelic attachment was significantly reduced after Rab6a knockdown (38.2 ± 4.1 vs. 75.1 ± 3.0% control, *p* < 0.05), whereas the percentages of undefined attachment (Fig. 4B, chromosomes 5), loss attachment (kinetochores unattached to either pole; Fig. 4B, chromosomes 6 and 7) and merotelic attachment (one kinetochore attached to both poles; Fig. 4B, chromosomes 8) in Rab6a-siRNA oocytes were all accordingly increased in comparison to control cells (Fig. 4C). These attachments are regarded as errors that must be corrected because chromosome missegregation would be produced if they persisted until anaphase. Collectively, the results are indicative of, in Rab6a-depleted oocytes, the erroneous K-MT attachments could result in the meiotic defects observed in our experiments.

### Rab6a knockdown provokes the spindle assembly checkpoint

**Figure 5:**
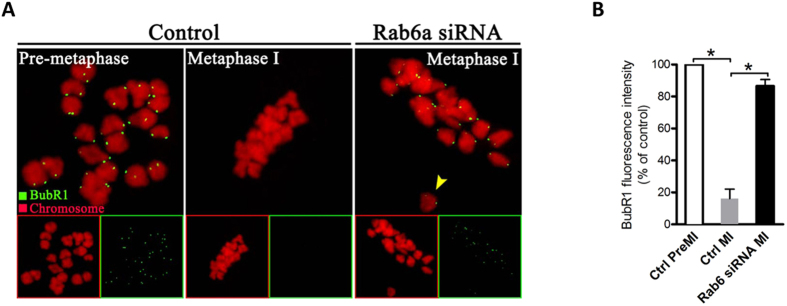
Rab6a knockdown provokes the spindle assembly checkpoint in oocyte meiosis. (**A**) Control and Rab6a-siRNA oocytes were labeled with anti-BubR1 antibody (green) and counterstained with PI to visualize chromosomes (red). Representative images of pre-metaphase I and metaphase I oocytes are shown. Arrowhead indicates the misaligned chromosomes in Rab6a-depleted oocytes. (**B**) Quantification of BubR1 staining in control and Rab6a-siRNA oocytes. At least 30 oocytes were analyzed for each group. Error bars indicate ± SD. *p < 0.05 vs controls.

Cells have a sophisticated safety mechanism known as the spindle assembly checkpoint (SAC) to ensure that chromosomes have time to correctly line up on the spindle before the cell can divide^23^. To successfully complete meiosis, chromosomes in oocyte must congress to the spindle equator and generate amphitelic K-MT attachments. In the absence of such attachments oocyte will delay meiotic exit^24^. The mechanism that monitors and responds to K-MT attachment is the SAC; and two proteins, called Bub1 and BubR1, play an essential role in this process^23^,^25^. Considering the K-MT misattachments and meiosis I block in Rab6a-depleted oocytes, we reasoned that these defects may arise from the SAC activation. To test this possibility, we analyzed the SAC activity during oocyte meiosis by immunolabeling of BubR1, which is an integral part of checkpoint complex and in its absence, SAC control is lost^26^. As shown in Fig. 5A, in normal oocytes, BubR1 was localized to unattached kinetochores at pre-metaphase I, and almost lost at metaphase I when kinetochores are properly attached. However, when Rab6a was abated, BubR1 expression on the kinetochores was markedly increased to approximately normal levels in meiosis I-arrested oocytes (Fig. 5A,B), indicative of the activation of SAC. Together, these results imply that the effects of Rab6a on meiotic structures and progression are likely to be mediated through SAC signaling in oocytes.

## Discussion

Rab family GTPases have been implicated in vesicle formation, vesicle delivery along cytoskeleton elements and docking at target membranes through the recruitment of effectors^27^. Here we discover a novel role for Rab6a during meiosis: the involvement in chromosome/spindle organization and metaphase/anaphase transition in mammalian oocytes.

Our cytological analysis revealed the failure of chromosome congress and spindle assembly in oocytes when Rab6a function was altered. In particular, lagging chromosomes were frequently observed in Rab6a-siRNA anaphase oocytes. Karyotypic analysis also confirmed the increased aneuploidy rate of these oocytes (Figs 2 and 3). Accurate alignment and segregation of homologous chromosomes or sister chromatids is a crucial event in meiosis. Chromosome movement depends on the establishment of physical and biochemical interactions between spindle microtubules and specialized chromosomal regions, the kinetochores^28^. Any errors in this process may result in aneuploid egg formation, which causes early embryo death, spontaneous abortion and genetic diseases^29^,^30^. In line with this notion, we found that Rab6a knockdown apparently impaired the K-MT attachments in meiotic oocytes (Fig. 4). These data collectively indicate that loss of Rab6a function could compromise K-MT interactions, whereupon lead to the chromosome misalignment and aneuploidy production in oocytes. In addition, we found that, when Rab6a was abated, the microtubule network was impaired in fully-grown immature oocytes and the actin cap failed to form in metaphase oocytes (unpublished data), indicating that Rab6a plays an important role in the maintenance of cytoskeletal structure during oocyte maturation. On the other hand, these findings imply that disorganization of microtubule and actin filament may contribute, at least in part, to the symmetric division (Fig. 1E) and spindle defects (Fig. 2B) we observed in Rab6a-siRNA oocytes.

Moreover, another important phenotype is that Rab6a-depleted oocytes were blocked in metaphase (Fig. 1). It has been extensively reported that the kinetochore functions as a structural platform and as a signaling hub that coordinates chromosome attachment, SAC activity, and cell cycle progression from metaphase to anaphase^31^. The SAC is an evolutionarily conserved regulatory mechanism that responds to the presence of improper K-MT attachments and inhibits cell progression to prevent errors in chromosome separation^32^. A large body of evidence has shown that the SAC exists in mouse oocytes and is able to recognize unattached kinetochores as in mitosis^24^. Importantly, our confocal scanning showed that BubR1 is constantly present in Rab6a-siRNA oocytes even at metaphase stage (Fig. 5). By using a conditional knockout approach, Touati *et al.,* recently demonstrated that meiotic SAC was defective in *BubR1* null oocytes, and accordingly, meiosis I was accelerated and chromosomes were not aligned at the metaphase plate^26^. Interestingly, they also found that BubR1 is required for the establishment of stable spindles in oocytes. The above observations strongly suggest that the activation of SAC signal is probably a major factor contributing to the meiosis I arrest in Rab6a-depleted oocytes.

On the basis of our findings, two important questions are raised: how Rab6a affects the K-MT attachments and SAC signaling in oocyte meiosis, and what proteins are the potential effectors mediating this pathway? Although the definite molecular mechanism remains unknown, genetic and biochemical assays have revealed some critical clues during these processes. For example, a direct role of the dynein/dynactin complex in the transport of several kinetochore proteins and the spindle checkpoint inactivation has been found^33^,^34^. Rab6a′ is proposed to be able regulate the dynamics of the dynein/dynactin complex at the kinetochores and consequently trigger the spindle checkpoint through interaction with p150^Glued^ and GAPCenA^35^. Future experiments aimed to characterize the interaction between Rab6a and kinetochore proteins will help to clarify the above questions. Regardless, due to the limitation of oocyte number and technical reason, we have not yet been able to directly screen the potential targets of Rab6a in mouse oocytes.

In conclusion, our data support a model: Rab6a knockdown in oocytes may compromise the interaction between kinetochore and microtubule, which in turn leads to the activation of SAC signal, and, as a result, chromosome misalignment and spindle defects are established during meiosis, causing the production of aneuploid eggs and persistent defects in embryos.

## Materials and Methods

All chemicals and reagents were obtained from Sigma unless otherwise stated. ICR mice were used in this study. All experiments were approved by the Animal Care and Use Committee of Nanjing Medical University and were performed in accordance with institutional guidelines.

### Antibodies

Rabbit polyclonal anti-Rab6a and Sheep polyclonal anti-BubR1 antibodies were purchased from Abcam (Cambridge, MA, USA; Cat#: ab95954 and ab28193); Human anti-centromere CREST antibody was purchased from Antibodies Incorporated (Davis, CA, USA; Cat#:15–234). Cy5-conjugated donkey anti-human IgG and FITC-conjugated donkey anti-goat IgG were purchased from Jackson ImmunoResearch Laboratory (West Grove, PA, USA; Cat#: 709-605-149 and 705-095-147). FITC-conjugated goat anti-rabbit IgG was purchased from Thermo Fisher Scientific (Rockford, IL, USA). Mouse monoclonal anti-β-actin antibodies and mouse monoclonal FITC-conjugated anti-α-tubulin antibodies were purchased from Sigma (St. Louis, MO, USA; Cat#: A5441 and F2168).

### Oocyte collection and culture

Female ICR mice (4–6 weeks) were sacrificed by cervical dislocation after intraperitoneal injections of 5 IU pregnant mare serum gonadotropin (PMSG) for 46 hours. Cumulus-enclosed oocytes were retrieved by manual rupturing of antral ovarian follicles. Fully-grown denuded oocytes were collected by removing cumulus cells with repeatedly mouth-pipetting. Oocytes were cultured in M16 medium under mineral oil at 37 °C in a 5% CO2 incubator for *in vitro* maturation.

### siRNA knockdown

Fully-grown immature oocytes were microinjected with Rab6a-targeting siRNA to knock down Rab6a proteins. siRNA was diluted with water to give a stock concentration of 1 mM, and 2.5 picoliter solution was injected. A siRNA negative control was injected as control. To facilitate the siRNA-mediated mRNA degradation, oocytes were arrested at GV stage in M2 medium containing 2.5 μM milrinone for 20 hours, and then cultured in milrinone-free medium for further experiments. Rab6a-siRNA sequence: 5′- GGAGCAACCAGUCAAUGAATT-3′; 5′-UUCAUUGACUGGUUGCUCCTT-3′ Control siRNA sequence: 5′-UUCUCCGAACGUGUCACGUTT-3′; 5′-ACGUGACACGUUCGGAGAATT-3′.

### Immunofluorescence and Confocal Microscopy

Oocytes were fixed with 4% paraformaldehyde in PBS (PH 7.4) for 30 minutes, permeabilized with 0.5% Triton X-100 for 20 minutes and then blocked in 1% BSA-supplemented PBS for 1 hour at room temperature. The processed samples were incubated overnight at 4 °C with primary antibodies as follows: anti-BubR1 antibody (1:250) and FITC-conjugated α-tubulin antibody (1:200). FITC- or Cy5-conjugated secondary antibodies were then applied for 1 hour at room temperature as appropriate. Chromosomes of oocytes were evaluated by costaining with propidium iodide (red) or Hoechst 33342 (blue) for 10min. Samples were examined under a laser scanning confocal microscope (LSM 700; Zeiss, Oberkochen, Germany).

For quantification of oocytes with meiotic defects, the gross morphology of spindle/chromosomes was assessed. To detect kinetochores, oocytes were labeled with human CREST auto-immune antibody (1:500) according to the protocol previously described^15^. To assess the kinetochore-microtubule (K-MT) attachments, oocytes were briefly chilled at 4 °C to induce depolymerization of non-kinetochore microtubules just prior to fixation. To measure the intensity of fluorescence, Image J software (NIH) was used as previously described^36^.

### Western blot analysis

A pool of 100 oocytes was lysed in Laemmli sample buffer containing protease inhibitor, and then subjected to 10% SDS-PAGE, resolved and electroblotted onto a PVDF membrane. Membranes were blocked in Tris-buffered saline containing 0.1% Tween 20 and 5% low fat dry milk for 1 hour and then incubated with anti-Rab6a antibody (1:1,000) overnight at 4 °C. After multiple washes in Tris-buffered saline containing 0.1% Tween 20 and incubation with anti-rabbit horseradish peroxidase linked antibody, the protein bands were visualized using an ECL Plus Western Blotting Detection System (GE Healthcare, Piscataway, NJ, USA). The membrane was then stripped and reblotted with anti-β-actin (1:5,000) antibody for loading control.

### Chromosome spread

Chromosome spreading was performed as previously described^37^. Oocytes were exposed to Tyrode’s buffer (pH 2.5) for to remove zona pellucidae, and then fixed in a drop of 1% paraformaldehyde with 0.15% Triton X-100 on a glass slide. Samples were labeled with CREST (1:500) for 1 hour to detect kinetochores, and chromosomes were counterstained with Hoechst 33342. Laser scanning confocal microscope was used to examine chromosome numbers in oocytes.

### Statistical Analysis

Data are presented as mean ± SD, unless otherwise indicated. Differences between 2 groups were analyzed by Student’s *t* test. Multiple comparisons between more than 2 groups were analyzed by 1-way ANOVA test using Prism 5.0. *P* < 0.05 was considered to be significant.

## Additional Information

**How to cite this article**: Hou, X. *et al.* Rab6a is a novel regulator of meiotic apparatus and maturational progression in mouse oocytes. *Sci. Rep.* **6**, 22209; doi: 10.1038/srep22209 (2016).
